# Heavy or healthy? Assessing menstrual bleeding and bleeding tendency in Dutch adolescents

**DOI:** 10.1016/j.rpth.2025.103235

**Published:** 2025-10-25

**Authors:** Anne de Vaan, Habibe Özcan, Nicole B. Burger, Robert A. de Leeuw, Judith A.F. Huirne, Nehalennia van Hanegem, Karin P.M. van Galen

**Affiliations:** 1 Center for Benign Hematology, Thrombosis and Hemostasis, van Creveldkliniek, University Medical Center, University Utrecht, Utrecht, the Netherlands; 2 Department of Obstetrics and Gynaecology, Amsterdam Reproduction and Development Research Institute, Amsterdam UMC, location AMC and VUmc, Amsterdam, the Netherlands; 3 Department of Obstetrics and Gynaecology, University Medical Centre Utrecht, Utrecht, the Netherlands

**Keywords:** adolescent, inherited blood coagulation disorders, menorrhagia, menstruation, software

## Abstract

**Background:**

Heavy menstrual bleeding (HMB) is common in adolescents and may indicate a bleeding disorder. The pictorial blood loss assessment chart (PBAC) defines HMB as a score ≥150, while an elevated self-bleeding assessment tool (self-BAT) score identifies people at risk for a bleeding disorder (age 12-17 years ≥3 and 18-21 years ≥6). PBAC and self-BAT scores from the general menstruating population are, however, unknown.

**Objectives:**

To assess the prevalence and association of HMB and elevated self-BAT scores in adolescents.

**Methods:**

Adolescents (aged 12-21 years) using the Menstruation Education Calendar application were included if they had completed the self-BAT and ≥1 PBAC. The Menstruation Education Calendar application is a mobile Health intervention and facilitates monitoring of menstrual blood loss. Logistic regression was used to assess the association between PBAC ≥150 and elevated self-BAT scores.

**Results:**

Between 2023 and 2024, 330 adolescents completed the workup. Of the participants, 22.1% (n=73/330) had a PBAC score ≥150, and 16.4% (n = 54/330) had an elevated self-BAT score. PBAC scores ≥150 were associated with an increased risk for an elevated self-BAT (odds ratio [OR], 2.48; 95% CI, 1.32-4.64). PBAC score ≥150 was equally prevalent in adolescents aged between 12 and 17 and 18 and 21 years (OR 0.70; 95% CI, 0.37-1.31). Adolescents aged 18 to 21 years had a lower risk for an elevated self-BAT compared with those aged 12 to 17 years (OR, 0.11; 95% CI, 0.06-0.20).

**Conclusion:**

Of adolescents, 20% have HMB. In adolescents aged 12 to 17 years, the self-BAT cutoff score ≥3 is too sensitive, identifying ∼50% at risk for a bleeding disorder and strengthening the need for reassessing the self-BAT cutoff score in menstruating adolescents.

## Essentials

• The association between menorrhagia and abnormal bleeding phenotype in adolescents is unknown.
• Participants completed a pictorial bleeding chart and self-reported bleeding tool using an app.
• Menorrhagia was equally prevalent in adolescents aged 12 to 17 years and 18 to 21 years.
• Menorrhagia was associated with abnormal bleeding in adolescents aged 12 to 17 years but not those aged 18 to 21 years.

## 1 Introduction

Menstrual complaints are widespread in adolescents, with one of the most common being heavy menstrual bleeding (HMB) [1,2]. The prevalence of HMB among adolescents varies between 12% and 37% [3,4]. HMB has various underlying causes, such as gynecologic disorders and bleeding disorders [5]. Bleeding disorders have been identified in 10% to 62% of adolescents with HMB [6]. Furthermore, the incidence of bleeding disorders in adolescents with HMB is higher than that in adults with HMB [7].

Several instruments are available to assess both HMB and bleeding disorders. The most widely used method to assess menstrual blood loss is the pictorial blood loss assessment chart (PBAC) [8]. A PBAC score ≥150 per menstrual cycle is defined as HMB and corresponds with blood loss of >80 mL according to the alkaline hematin method [8,9]. It is worth mentioning that the PBAC is usually used in research settings and not in common practice in standardized care in the Netherlands. A validated instrument used to identify a person at risk for a bleeding disorder is the International Society of Thrombosis and Haemostasis Bleeding Assessment Chart (ISTH-BAT) [10]. The ISTH-BAT contains a menstruation-specific domain, with a score ≥3 indicating severe menstrual bleeding with impact on life [10,11]. While the ISTH-BAT is usually administered during a hematologic consult, a more simplified version is available that can be completed by individuals (self-BAT) [12].

Despite using validated instruments, providing appropriate care and conducting a diagnostic workup for adolescents with HMB remains challenging. First, many adolescents are unaware that they are experiencing HMB and do not seek medical help, leading to diagnostic delays [13,14]. For example, >60% of adolescents with self-reported normal menstrual bleeding have HMB [15]. Second, when they do seek help, hormonal contraceptives are often prescribed without further evaluation [16]. Although hormonal treatment may alleviate symptoms, it may not address the underlying cause. This is especially problematic for individuals with an underlying bleeding disorder, who often experience hemostatic challenges later in life during surgery or childbirth [17].

Ideally, the self-BAT should be used in adolescents with HMBs assessed with the PBAC to identify those at risk for an underlying bleeding disorder. However, it remains unknown how self-BAT scores correlate with PBAC scores, specifically in menstruating adolescents. This study aims to assess the prevalence of increased total self-BAT scores and its association with objectified HMB by elevated PBAC scores in Dutch adolescents. The secondary aim is to assess the association between HMBs and the specific menstruation domain of the self-BAT.

## 2 Methods

### 2.1 Study design

This prospective cohort study investigated menstrual blood loss and the prevalence of increased bleeding tendency in adolescents. It involved the self-BAT and the utilization of the Menstruatie Educatie Kalender (Menstruation Education Calendar) application (MEK app) for ≥1 menstrual period. This study was part of a study investigating menstrual complaints and their impact registered in ClinicalTrials.gov (NCT06921629). It was approved by the Medical Ethics Review Committee of VU University Medical Center (W21_307#21.341).

### 2.2 Participants

Participants were recruited between May 2023 and October 2024 from social media (Facebook, Instagram, and TikTok), biology classes, and gynecology outpatient clinics regardless of their reason for visiting. Inclusion criteria were postmenarchal adolescents aged between 12 and 21 years (in accordance with the Dutch Youth Institutes [18]) with monthly vaginal bleeding, having a smartphone with the Android or iOS operating system, and the ability to communicate in Dutch, as the MEK app is only available in Dutch.

Participants self-screened for eligibility and registered online. To avoid duplicates, each email address could only be used once. Participants aged 12 to 16 years had to provide parental consent. After registration and completing a baseline questionnaire in the MEK app, the subject was additionally screened for eligibility. Those who did not menstruate or did not adhere to the other eligibility criteria were removed from the analyses.

### 2.3 Instruments

Upon registering for the study, participants received a questionnaire comprising baseline characteristics and the self-BAT. Baseline characteristics consisted of age, education level, ethnicity, parity, menarche, use of contraceptive pills, use of an intrauterine device, use of anticoagulants, bleeding disorders, if menstrual complaints were normal, and if they visited a physician because of their menstrual complaints. For readability, when we report oral contraceptives and intrauterine devices together, we refer to them as contraceptives.

#### 2.3.1 The self-BAT

The self-BAT consists of 14 bleeding domains, including a menstruation domain, with each domain being awarded points from 0 (no/trivial bleeding) to 4 (bleeding that required advanced hemostatic interventions such as coagulation factor concentrate) [10]. For this study, we use 2 outcomes for the self-BAT: the total score and the menstruation score, based on the menstruation-only domain. The validated abnormal total self-BAT scores used were ≥3 for children ≤18 years old and ≥6 for menstruating adults [19]. A score ≥3 on the self-BAT menstruation domain is defined as severe menstrual bleeding according to the self-BAT [10,11]. Only complete self-BAT records were included in the analysis. The total self-BAT score and self-BAT menstruation scores were calculated. Participants with abnormal self-BAT scores were dichotomized based on the predefined age cutoffs: 12 to 17 years ≥3, and 18 to 21 years ≥6 [10,19]. At the end of the self-BAT, an optional open question for remarks was added. Participants with abnormal self-BAT scores were contacted through email and advised to visit a general practitioner (GP) for consultation since we believe it is our responsibility as clinical researchers to inform subjects in case of abnormal test results. In the Netherlands, the GP acts as a gatekeeper, meaning that patients must first consult their GP before being referred to secondary or tertiary care. We gave the participant a letter that they should bring to the GP that explained the study and included our recommendations on bloodwork (activated partial thromboplastin time, prothrombin time, platelet count, and von Willebrand factor assays) if the GP had a high suspicion for a bleeding disorder after consulting the participant. After 3 months, we contacted the participants again, asking them if they had gone to their GP or a specialist, if any additional testing had been performed, and what the outcome was. It is not the aim of the study to assess the prevalence of a bleeding disorder.

#### 2.3.2 MEK app

After completing the self-BAT, participants used the MEK app daily for ≥1 period. The MEK app is a mobile Health (mHealth) application for evaluating menstrual complaints. Its development followed the waterfall methodology, a linear approach in which software development follows a sequence of activities, with each phase producing a set of requirements that serves as input for the next phase’s design [[20], [21], [22]]. Previous evaluation showed that both adults and adolescents can use the MEK app [23].

The MEK app is a calendar that facilitates daily monitoring of pain, spotting, the amount of blood loss using PBAC, and the impact of these complaints on daily life activities. The MEK app contains pictograms of saturated menstruation products for comparison with their used products to ensure adequate PBAC entry (Supplementary Figure). If participants recorded >1 cycle in the MEK app, the average PBAC score over the different months was calculated. Cycles for PBAC completion could be sequential or random.

### 2.4 Subgroup analysis

The self-BAT does not differentiate between children and adolescents and uses only an age-dependent cutoff of ≥18 years for adults. This differs from that of the Dutch Youth Institute, which states adolescence is until the age of 21 years. To assess the prevalence of increased self-BAT scores in the Dutch adolescent population, subgroup analyses on the age groups 12 to 17 and 18 to 21 years were performed.

### 2.5 Sample size

As this longitudinal study involved a sample from the general population, it was not feasible to calculate an exact sample size in advance. A pragmatic approach was therefore adopted, whereby participants were recruited over a defined inclusion period. In the literature, approximately 30% of adolescents with HMB had abnormal self-BAT scores, matching the estimated prevalence of an underlying bleeding disorder in adolescents with HMB [[24], [25], [26]]. With 80% power and an alpha of 5%, a sample size of at least 323 adolescents is needed. Accounting for 10% loss to follow-up or an incomplete workup, a total number of 356 participants are required.

### 2.6 Statistical analysis

We performed descriptive analysis for baseline characteristics, PBAC, and self-BAT scores. Q-Q plots and boxplots were used to determine the distribution of continuous data. Normally distributed data are reported as mean and SD, and nonnormally distributed data is reported as median and IQR. Categorical data are depicted in absolute numbers and frequencies (percentage). We performed 2 subgroup analyses: one based on HMB according to a PBAC score <150 or ≥150, and one based on the age-dependent cutoff for elevated self-BAT scores (12-17 years and 18-21 years, based on the age at registration). Statistical difference between the subgroups was assessed by the Student’s *t*-test for normally distributed data, Mann–Whitney U-test for nonnormally distributed data, and chi-squared test for categorical data. Logistic regression was used to calculate odds ratios (ORs) with a 95% CIs to assess the risk of a self-BAT score above the age-dependent cutoff in participants with a PBAC score ≥150. The model was adjusted for contraceptive use. *P* values <.05 were considered statistically significant.

## 3 Results

A total of 921 participants were enrolled. Most participants (55%) were enrolled through Instagram, followed by 24% via TikTok, 11% through family or acquaintances, 7% via outpatient clinics, 2% through schools, and 1% via Facebook. Of the 921 enrolled participants, 142 were excluded because they did not download the MEK app, 46 were excluded due to age >21 years, 42 were excluded due to unknown age, and 9 due to duplicate registration (see Figure). Of the remaining 682 participants, 309 did not complete the self-BAT, 24 did not use the app, and 19 had amenorrhea. This resulted in 330 participants who recorded ≥1 menstrual period in the MEK app after completing the self-BAT.

**Figure:**
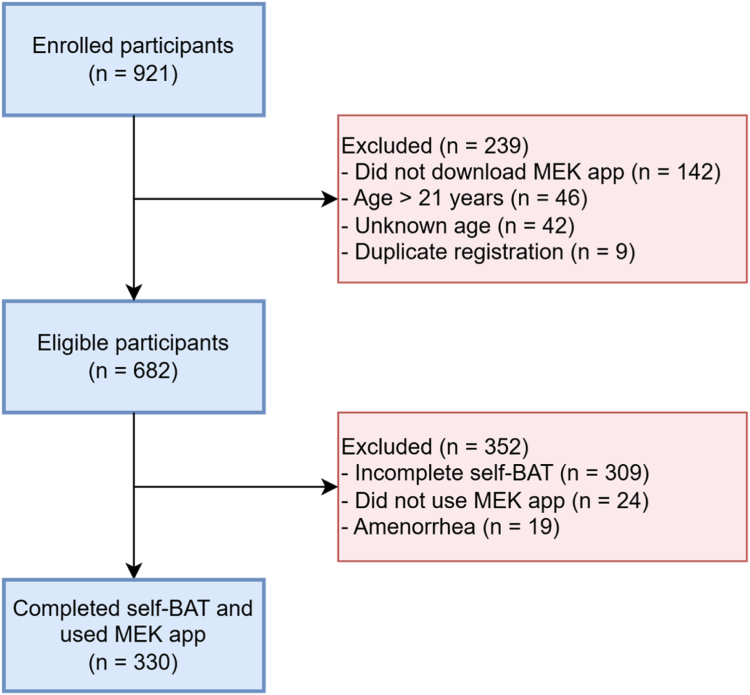
Overview of the inclusion and selection process. BAT, Bleeding Assessment Tool; MEK, Menstruation Education Calendar; n, number.

The majority of the participants were aged 18 to 21 years old (*n* = 268, 81.2%). All participant were nulliparous. The baseline characteristics are displayed in Table 1.

**Table 1:** Baseline characteristics.

Variable	All (*N* = 330)
Age, y, median (IQR)	19.8 (18.5-20.8)
Age 12-17 y, *n* (%)	62 (18.8)
Ethnicity^a^, *n* (%)	
European	298 (90.3)
Asian	8 (2.4)
Middle Eastern	3 (0.9)
Turkish	7 (2.1)
African	7 (2.1)
Other^b^	7 (2.1)
Level of education based on ISCED, *n* (%)	
Primary education	25 (7.6)
Lower secondary education	58 (17.6)
Upper secondary education	191 (57.9)
Post-secondary nontertiary education	0 (0)
Short-cycle tertiary education	0 (0)
Bachelor’s degree	53 (16.1)
Master’s degree	3 (0.9)
Familial bleeding disorder, *n* (%)	
Yes	15 (4.5)
No	219 (66.4)
Unknown	96 (29.1)
Consulted a physician for menstrual complaints, *n* (%)	127 (38.5)
General practitioner	86 (67.7)
Gynecologist	41 (32.3)
Diagnosis for menstrual complaints, *n* (%)	
Not investigated	203 (61.5)
Gynecological diagnosis^c^	18 (5.5)
Other^d^	1 (0.3)
Still in diagnostic process	11 (3.3)
No diagnosis^e^	97 (29.4)

Results are self-reported by participants.

ISCED, International Standard Classification of Education.

a As reported by the participant.

b Includes Antillean and Afghan ethnicity.

c Includes polycystic ovarian syndrome (*n* = 11), endometriosis (*n* = 4), adenomyosis (*n* = 2), and premenstrual syndrome (*n* = 1).

d Includes stress (*n* = 1).

e Consulted a physician but did not receive a diagnosis.

Irregular menstrual cycles were self-reported in 35.2% (*n* = 116/330) participants, mostly by the younger adolescents 18 to 21 years old (67.2%, *n* = 78/116). The majority (61.5%) of the participants had not consulted a physician for menstrual complaints. Those who did (n=127) mostly consulted their GP, and in the majority of the participants, a diagnosis was not made (*n* = 97/127, 76.4%). Overall, 54.5% (*n* = 180) considered their menstrual period normal, and 73.3% (*n* = 242) perceived the amount of menstrual blood loss as normal. There was 1 participant who used acetylsalicylic acid when asked about anticoagulant use.

The median age for menarche was 12 years (IQR, 11-13 years). Approximately 23% of the participants used some form of contraceptives, mostly for a combined purpose of contraception, dysmenorrhea, and HMB. There were 196 participants (59.4%) that completed 2 valid PBACs. Mean PBAC score was 115.2 (SD 92.8), and 22.1% (*n* = 73/330) had HMB according to a PBAC score ≥150. Other data on the menstrual cycle are reported in Table 2.

**Table 2:** Menstruation and self-BAT data.

Variable	All (*N* = 330)
Menarche, y, median, (IQR)	12.0 (11.0-13.0)
Gynecological age^a^, y, median, (IQR)	7.3 (5.6-8.6)
Menstrual cycle duration, d, median, (IQR)	29.0 (27.5-33.0)
Menstrual bleeding duration, d, median, (IQR)	5.5 (4.5-6.5)
Contraceptive use, *n* (%)	78 (23.6)
Type of contraceptives used, *n* (%)	
Oral contraceptive	60 (18.2)
Hormonal intrauterine device	11 (3.3)
Copper intrauterine device	7 (2.1)
Reason for contraceptive use, *n* (%)	
Contraception	25 (32.1)
Heavy menstrual bleeding	2 (2.6)
Dysmenorrhea	9 (11.5)
Combined purpose^b^	42 (53.9)
PBAC	
PBAC score, median (IQR)	99.5 (55.4-140.0)
No contraceptive users	106.3 (61.9-146.9)
Contraceptive users	75.3 (40.4-118.4)
PBAC score ≥150, *n* (%)	73 (22.1)
No contraceptive users	61 (83.6)
Contraceptive users	12 (16.4)
Perceived menstrual blood loss as abnormal, *n* (%)	86 (26.7)
Self-BAT	
Total self-BAT score, median (IQR)	3 (2-4)
Self-BAT scores above age-dependent cutoff^c^, *n (%)*	54 (16.4)
Self-BAT menstruation domain score, median (IQR)	2 (1-3)
Self-BAT menstruation domain distribution, *n* (%)	
0	27 (8.2)
1	104 (31.5)
2	68 (20.6)
3	130 (39.4)
4	1 (0.3)

Self-BAT menstruation domain scores ≥3 indicate severe menstrual bleeding.

BAT, bleeding assessment tool; HMB, heavy menstrual bleeding; PBAC, pictorial bleeding assessment chart.

a Gynecological age is the age minus age at menarche.

b Includes contraception/dysmenorrhea/HMB (*n* = 15), contraception/dysmenorrhea (*n* = 9), contraception/HMB (*n* = 1), dysmenorrhea/HMB (*n* = 17).

c ≥3 for 12 to 17 years, ≥6 for 18 to 21 years.

The mean self-BAT score was 3.0 (SD 1.8), with a total range from 0 to 15. Most points were scored on the menstruation and epistaxis domain. In total, 131 participants reported a self-BAT menstruation domain score ≥3, indicating severe bleeding. The participants who perceived that their menstrual blood loss was abnormal (*n* = 86/328) had almost a 6 times higher chance for severe menstrual bleeding according to the self-BAT menstruation domain (OR, 5.8; 95% CI, 3.4-9.9) than those who perceived their blood loss as normal (*n* = 242/328).

More participants with a PBAC score ≥150 had a self-BAT higher than their age-dependent cutoff (OR, 2.46; 95% CI, 1.32-4.64) and had a self-BAT menstruation domain score ≥3 (Table 3). Participants with a PBAC score <150 more frequently reported that they perceived their menstrual blood loss as normal (OR, 3.40; 95% CI, 1.96-5.91).

**Table 3:** Self-BAT outcomes based on the PBAC cutoff of 150.

Variable	PBAC ≥150 (*n* = 73)	PBAC <150 (*n* = 257)	OR (95% CI) or *P*
Age, y, median (IQR)	19.5 (18.2-20.9)	19.9 (18.5-20.8)	.29
PBAC score, median (IQR)	213.0 (167.8-270.8)	80.0 (46.5-111.8)	<.001^a^
Contraceptive use, *n* (%)	12 (16.4)	66 (25.7)	0.57 (0.29-1.12)
Perceived menstrual blood loss as normal, *n* (%)	39 (53.4)	203 (79.0)	3.40 (1.96-5.91)^a^
Self-BAT score median (IQR)	4 (3-5)	3 (2-4)	<.001^a^
Self-BAT ≥ age-dependent cutoff^b^, *n* (%)	20 (27.4)	34 (13.2)	2.48 (1.32-4.64)^a^
Self-BAT menstruation domain ≥3, *n* (%)	44 (60.3)	87 (33.9)	2.97 (1.74-5.06)^a^
Self-BAT menstruation domain score, median (IQR)	2 (2-3)	2 (1-3)	<.001^a^

Self-BAT menstruation domain scores ≥3 indicate severe menstrual bleeding.

OR, odds ratio; PBAC, pictorial bleeding assessment chart; self-BAT, self-bleeding assessment tool.

a *P* < .05 and 95% CI of OR >1.0 considered statistically significant difference.

b ≥3 for age 12 to 17 years, ≥6 for age 18 to 21 years.

### 3.1 Contraceptive use

Participants who used contraceptives had a significantly lower median PBAC score than those who did not use contraceptives (*P* < .001, Table 2). When considering only the participants not using contraceptives (*n* = 252), elevated self-BAT scores occurred more often in those with a PBAC score ≥150 (*n* = 14/61, 30.0% vs *n* = 24/191, 12.7%; *P* = .048). Contraceptive use occurred similarly between those with and without a PBAC score ≥150 (25.7% vs 16.4%; OR 0.57, 95% CI, 0.29-1.12). Adjusting the model for contraceptive use did not significantly change the risk of a self-BAT score higher than the age-dependent cutoff (OR, 2.62; 95% CI, 1.38-4.94).

### 3.2 Subgroup analysis based on age

We divided the total cohort in 2 subgroups based on the ISTH-BAT cutoff for age (<18 years). There were 62 participants aged 12 to 17 years, and 268 participants aged 18 to 21 years. Table 4 displays self-BAT outcomes based on age.

**Table 4:** Self-BAT outcomes based on the self-BAT cutoff for age.

Variable	12-17 y (*n* = 62)	18-21 y (*n* = 268)	OR (95% CI)	*P*
PBAC score, median (IQR)	108.3 (70.0-156.6)	98.0 (52.1-132.1)	n/a	.06
PBAC ≥150, *n* (%)	17 (27.4)	56 (20.9)	0.70 (0.37-1.31)	n/a
Contraceptive use, *n* (%)	10 (16.1)	68 (25.4)	1.77 (0.85-3.67)	n/a
Self-BAT score, median (IQR)	2 (2-3)	3 (2-4)	n/a	.11
Self-BAT above age-dependent cutoff, *n* (%)	30 (48.4)	24 (9.0)	0.11 (0.06-0.20)	n/a
Self-BAT menstruation score ≥3^a^, *n* (%)	17 (27.4)	114 (42.5)	1.96 (1.07-3.60)	n/a
Self-BAT menstruation score, median (IQR)	1.5 (1-3)	2 (1-3)	n/a	.03

Self-BAT menstruation domain scores ≥3 indicate severe menstrual bleeding. The subgroup 12 to 17 years was the reference group.

BAT, bleeding assessment tool; OR, odds ratio, n/a, not applicable; PBAC, pictorial bleeding assessment chart.

a ≥3 for age 12 to 17 years, ≥6 for age 18 to 21 years.

Overall, there was a nonsignificant trend of slightly higher PBAC scores in the participants aged 12 to 17 years compared with those aged 18-21 years (108 vs 98, *P* = .06). Contraceptive use was not statistically different between the 2 groups. The prevalence of HMB according to the PBAC score was comparable (OR, 0.70; 95% CI, 0.37-1.31). Participants between 18 and 21 years old had a much lower chance of having a total self-BAT score higher than their age-dependent cutoff (self-BAT ≥6) compared to those aged 12 to 17 (cutoff self-BAT score ≥3). In contrast, the chance of severe menstrual bleeding according to the self-BAT menstruation domain was higher in the 18 to 21 year-old participants (OR, 1.96; 95% CI, 1.07-3.60). This indicates that the increased risk for an abnormal self-BAT score in the 12 to 17 year-old group appears independent of the self-BAT menstruation domain.

Of the 54 participants with elevated self-BAT scores, 29 (53.7%) replied to our emails. Most of the nonresponders were in the 12 to 17 year-old group (66.6%, *n* = 20/30). The replies of the responders are summarized in Table 5.

**Table 5:** Follow-up outcomes of participants with abnormal self-BAT scores.

Variable *n* (%)	Participant with elevated self-BAT scores that replied to the follow-up emails (*N* = 29)
Did not visit a physician	10 (34.5)
Reasons to not visit a physician	
HMB runs in the family	1 (10.0)
Previous bleeding disorder testing	1 (10.0)
Not specified	8 (80.0)
Visited a physician	19 (65.5)
Physician consulted	
General practitioner	14 (73.7)
Hematologist	2 (10.5)
Gynecologist	3 (15.8)
Laboratory testing	11 (57.9)
Bleeding disorder diagnosis^a^	2 (10.5)
Therapeutic management for HMB	5 (26.3)
Hormonal oral contraceptive	2 (10.5)
Hormonal intrauterine device	2 (10.5)
Tranexamic acid	1 (53)

BAT, bleeding assessment tool; HMB, heavy menstrual bleeding.

a *n* = 1, von Willebrand disease type 1, and *n*=1 bleeding disorder of unknown cause.

## 4 Discussion

In this study, we determined the prevalence of HMB as assessed by the PBAC and the prevalence of abnormal self-BAT score in the general adolescent population aged 12 to 21 years old. One in 5 adolescents had HMB based on PBAC score ≥150. The occurrence of abnormal self-BAT scores was 16% according to the validated age-dependent cutoff. Adolescents with a PBAC score ≥150 had a higher risk for elevated self-BAT and self-BAT menstruation domain scores compared with those with a normal PBAC score. Subgroup analyses based on the self-BAT cutoff for adolescents (<18 years) revealed that, even though PBAC scores and the prevalence of HMB are comparable to those of adolescents ≥18 years, elevated total self-BAT occurred 5 times less often in the 18 to 21 year-old group. This is in line with a higher age-dependent self-BAT cutoff level. Of the menstruating adolescents aged 12 to 17 years, almost half had an increased total self-BAT score ≥3.

The observed high prevalence of HMB (20%) aligns with previously reported prevalence rates, though estimates vary widely (4.8%-37%) [27]. Approximately 24% of the participants used contraceptives for various reasons. Although contraceptive users had lower PBAC scores, they did not affect the likelihood of an abnormal self-BAT score in those with HMB, suggesting that the PBAC and self-BAT can be administered in adolescents using contraceptives and that it is important to consider a possible bleeding disorder in adolescents that continue to experience HMB despite the use of hormonal contraceptives. Notably, 40% of the adolescents reported severe menstrual bleeding on the self-BAT menstruation domain (menstruation score ≥3), twice as high as the ‘objectified’ HMB frequency according to the PBAC. This highlights a discrepancy between measured and self-reported severity of menstrual bleeding. One explanation is that the self-BAT menstruation domain may encompass other factors of menstruation, such as absence from school due to menstrual pain, or misinterpretation of questions. Additionally, our study shows that individual perception of menstrual bleeding varies and that the perception of abnormal menstrual blood loss leads to more severe bleeding being reported on the self-BAT. This underscores the need for health care providers to reassess the self-BAT menstruation domain to better align with objective measures of HMB such as the PBAC.

Subgroup analysis based on a PBAC score of 150 revealed that a PBAC score ≥150 is associated with elevated total self-BAT and self-BAT menstruation domain scores. This highlights the association between HMB and an increased risk of bleeding disorders. It would therefore be beneficial to administer the self-BAT to adolescents with a PBAC score ≥150. In clinical practice, however, menstruating adolescents undergoing diagnostic workup with the self-BAT often do not have a documented PBAC score. Furthermore, 34% of the participants with a PBAC score <150 reported severe bleeding on the self-BAT menstruation domain (menstruation score ≥3). This discrepancy suggests that either the self-BAT menstruation domain is too sensitive or too unclear for adolescents without the assessment of a physician. This is strengthened by earlier research that reported higher scores on the self-BAT than the ISTH-BAT in >10% of the cases [[26], [27], [28]]. Additionally, previous research showed similar menstruation-specific scores in adolescents with HMB with and without a bleeding disorder [28]. We suggest that both the PBAC and the self-BAT should be completed during the diagnostic workup in menstruating adolescents with HMB complaints, with the self-BAT responses being verified by a physician.

When subdividing the cohort based on the age-dependent cutoff for the self-BAT (18 years), we found that PBAC scores and the proportion of participants with a PBAC score ≥150 were comparable in both subgroups. However, adolescents aged 12 to 17 years had an increased risk for an abnormal self-BAT score (approximately 50%), whereas this was not observed in the 19 to 21 years subgroup. This suggests that the self-BAT cutoff of ≥3 is probably too sensitive in menstruating adolescents <18 years old. Earlier research reported similar findings, proposing that a cutoff of ≥4 to 5 would be more suitable in adolescent girls after menarche and that the self-BAT scores increase with age [17,28,29]. The original validation cohort of the ISTH-BAT and self-BAT cutoffs for children included participants with a mean age of 9 years (premenarchal) including both biological males and females [19]. This approach assumes that adolescent females aged <18 years are comparable to adolescent males <18 years, which does not account for the impact of menstruation and potential pregnancy. One could argue whether a meaningful difference in bleeding phenotype exists between a menstruating 17-and 18-year-old. Additionally, misinterpretation of the self-BAT could occur more often in younger adolescents, leading to an overestimation of the self-BAT menstrual domain. We believe that a self-BAT cutoff based on bleeding challenges instead of age would be more appropriate, implying a cutoff ≥4 for menstruating adolescents who did not experience childbirth. This aligns with previous research, in which a self-BAT cutoff of ≥5 had a specificity of 94% in adolescents with HMB [28].

Notably, >50% of the participants with a PBAC score ≥150 reported in the baseline questionnaire that they perceived their menstrual blood loss as normal, highlighting a gap in awareness. This adds to the body of evidence that the definition of HMB is not known in the general adolescent population or that questionnaires on menstruation are unclear for the younger population [13,14,30]. Proper education on schools and through easily accessible mediums, such as apps and websites like ‘Let’s Talk Period’ could resolve this problem [31]. Moreover, awareness across primary health care providers on normal and abnormal menstruation patterns and awareness of the possibility of an underlying bleeding disorder need to increase to aid timely diagnosis of bleeding disorders in adolescents with HMB [16,32,33].

## 5 Strengths and Limitations

To our knowledge, this is the first study to measure the PBAC and self-BAT in adolescents in the general population. One of the strengths was the study design. The prospective cohort study design provided valuable insights into the menstrual blood loss of adolescents, as recorded daily in an easily accessible digital PBAC scoring app.

Since participants were primarily recruited through social media, this may have introduced a selection bias, with adolescents more likely to participate due to increased concerns or complaints about their menstruation. The relatively high proportion of participants who visited a physician for menstruation complaints [16], compared with the low prevalence of adolescents consulting a GP for HMB, may be an indication of this bias. The app allows entry of data on passed days, introducing possible recall bias. However, when comparing the occurrence of HMB in adolescents in this study to that of previous studies, the prevalence aligns. Due to the study design, it was not possible for a provider to validate the self-BAT. With the known discrepancy between the self-BAT and the ISTH-BAT, it is possible that the self-BAT scores were overestimated [34]. However, the self-BAT has been validated based on the provider-administered ISTH-BAT, although the study population was not similar to this study. The same holds for the self-reported data on bleeding disorder diagnosis and previous diagnostic testing outside of this study—verification was not possible and limited validity. Another limitation is that only a few participants with elevated self-BAT scores responded to our follow-up emails about diagnostic testing for a bleeding disorder. This was mostly in the younger age group. Perhaps sending text messages instead of emails and/or including their parents in the communication could improve responses to follow-up. Almost half of these respondents did not visit a physician for diagnostic testing for a bleeding disorder, prohibiting conclusions regarding the prevalence of underlying bleeding disorders in this population. In addition, in this study, we used a PBAC cutoff value of 150 to define HMB [9]. However, various cutoff values of the PBAC are used in the literature, and many use a cutoff value of 100 for abnormal blood loss [8]. This threshold is commonly applied to enhance the comparability of results across studies [35,36]. Since a PBAC cutoff value of 100 has also been marked as too sensitive, HMB may be overdiagnosed [35]. Last, with menstrual cups and menstruation underwear also being used nowadays, the question whether the PBAC is still the most suitable instrument for measuring menstrual blood loss could be questioned, as it has only been validated for tampons and sanitary pads.

## 6 Conclusion

One in 5 menstruating adolescents <21 years of age in the general population has HMB, as determined by a PBAC score ≥150. PBAC scores ≥150 are likely associated with an increased bleeding tendency according to self-BAT scores above the age-dependent cutoff. The association between PBAC scores ≥150 and the ISTH-BAT might be less pronounced since self-BAT scores are often higher than ISTH-BAT scores. In menstruating adolescents aged 12 to 17 years, a self-BAT cutoff of ≥3 to define abnormal bleeding is too sensitive, flagging ∼50% of them at risk for a bleeding disorder. This strengthens the need for investigating appropriate self-BAT cutoffs based on bleeding challenges instead of age.
