# Supplementary material for: Heavy or healthy? Assessing menstrual bleeding and bleeding tendency in Dutch adolescents
Source: Res Pract Thromb Haemost. 2025 Oct 25;9(8):103235. doi: 10.1016/j.rpth.2025.103235 (PMC12666817; doi:10.1016/j.rpth.2025.103235)
Supplement: Supplementary Material [file mmc1.docx]

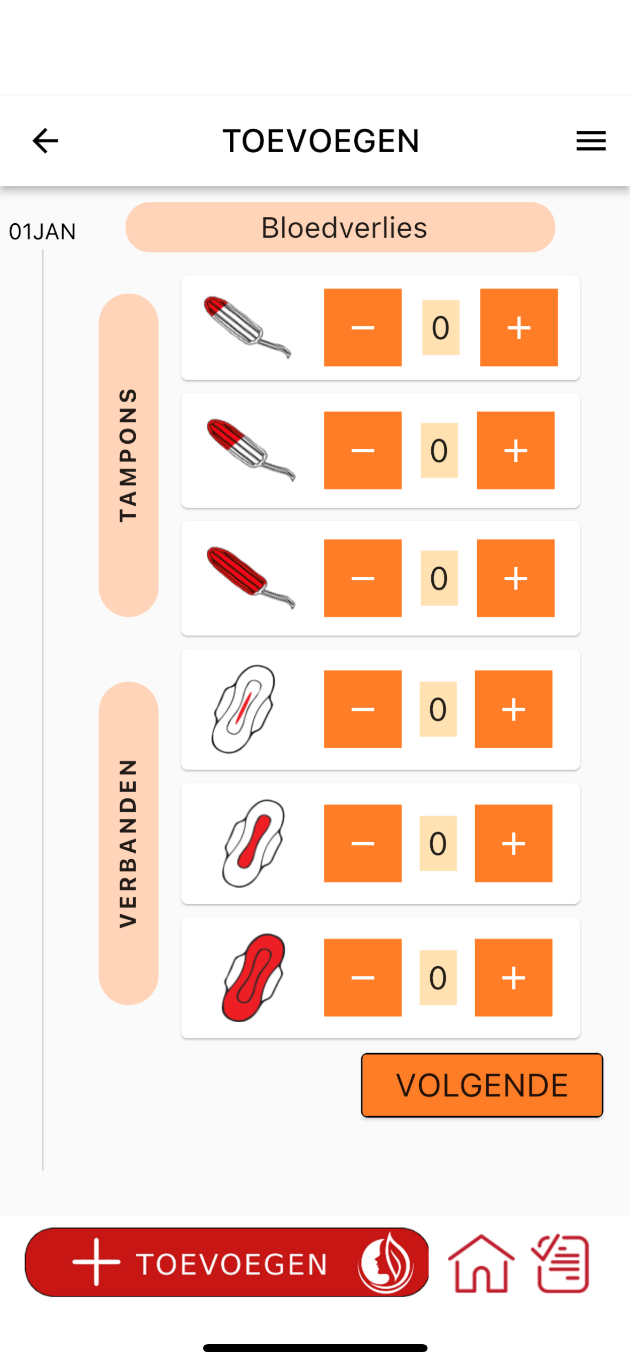
**Supplementary** Figure 1.

**Figure 1.** Screenshot of the Pictorial Bleeding Assessment Chart within the Menstruation Education Calendar Application.
